# Nephrotic syndrome and behavior problems in children

**DOI:** 10.4103/0019-5545.58903

**Published:** 2010

**Authors:** Imran Mushtaq, Mohammad Zafar Iqbal, John Kamara

**Affiliations:** Northampton CAMHS, 8-Notre Dame Mews, Northampton NN1 2BG, UK. E-mail: imranmushtaq@doctors.org.uk; 1 103-Collrge Avenue, Gillingham Kent ME7 5HX, UK

Sir,

Children with chronic physical illnesses are generally considered at increased risk for behavior difficulties. Illnesses not only affect their psychosocial development but also increase behavior problems in siblings and with added burden of disease on family life.[1] The literature on chronic illnesses provide evidence that conditions, such as insulin-dependent diabetes mellitus (IDDM),[2] cancer,[3] cystic fibrosis,[4] juvenile rheumatoid arthritis,[5] and asthma,[6] among others, are associated with increased psychopathology, including behavior problems in children. Nephrotic syndrome is one of the chronic illnesses of childhood that has significant association with behaviour problems, but there are not enough studies to study this in considerable details. Guha *et al.*,[7] are highly commendable for their efforts to support this notion, through their highly praiseworthy work on the topic. However, there are few points that need further clarifications from the authors, in order to make it easy for our understanding.

Firstly, authors could not find any association between use of corticosteroid therapy and behaviour disturbance, which is a significant finding considering the other studies providing the evidence for association and had been replicated in a number of studies. In this study, behavior disturbance was recorded in 34 (68%) of children. It is important to know how many of these children were on steroids at the time of assessment. As there is some evidence to suggest that corticosteroids significantly reduce the morbidity and mortality in children afflicted with leukemia, asthma, rheumatic diseases, and other inflammatory disorders, though often at the expense of behavioral problems, which may be either due to use of medication or the disease process (chronicity).[8]

Secondly, hyperkinesis at 32% (16 children) was over-repre sented in the nephrotic syndrome (NS) group. It is not entirely clear whether overactivity was considered to be only a symptom of behavior disturbance or diagnostic for attention deficit hyperactivity disorder (ADHD). If that is the case, then it may have therapeutic implications, knowing that ADHD may be under-recognized and under-diagnosed in low- and middle-income countries.

Thirdly, 50% of children in the NS group were not attending school and yet still the authors claim that the presence of behavior disturbance and inability to attend school is an important finding of the study. We could argue that non-attendance may be due to other factors, such as parents' socioeconomic status (84% in the study were from lower socioeconomic background), effects of illness itself, and parenting style e,g, being overprotective, rather than mere behaviour disturbance as a sole factor. It will be interesting to know how many children out of the 50% non-attenders that never attended school at all in the first instance.

Fourthly, there appears to be a statistically significant finding of the presence of behavior problem and in relation to the frequency of relapses per year, thus signaling the possibility of a recall bias. It also raises doubt as to whether the patients in remission could not have had any behavior problems, as the authors did not make this clear in the study.

Fifthly, it is again not entirely clear why behavioral disturbances at baseline were not considered in the exclusion criteria.

Sixthly, it appears that 6% of the NS group had learning disorder bearing in mind that mental retardation (MR) was one of the exclusion criteria. It is also important to note that learning difficulties and learning disability (MR) are often used synonymously, although they both can convey different messages or meanings.[9]

Finally, authors have not mentioned about the power of the study, which would have given us an indication of the significance of the study in relation to the results if they are to be considered valid.
